# Cardiac magnetic resonance parameters associated with successful conversion from a single ventricular to a one-and-a-half or biventricular circulation in patients with a hypoplastic right ventricle

**DOI:** 10.1186/s12968-023-00965-6

**Published:** 2023-09-28

**Authors:** Deepa Prasad, Jennifer Romanowicz, Puja Banka, Rebecca Beroukhim, Sunil J. Ghelani, Sitaram Emani, Andrew J. Powell

**Affiliations:** 1 Department of Cardiology, Boston Children’s Hospital, 300 Longwood Avenue, Boston, MA 02115 USA; 2 Department of Pediatrics, Harvard Medical School, Boston, USA; 3 Department of Cardiac Surgery, Boston Children’s Hospital, Boston, USA; 4 Department of Surgery, Harvard Medical School, Boston, USA; 5 Present Address: Banner Children’s at Desert Medical Center, University of Arizona College of Medicine-Phoenix, Phoenix, USA; 6 Present Address: Merck & Co., Inc, Rahway, NJ USA

**Keywords:** Cardiac magnetic resonance, Hypoplastic right ventricle, One and half ventricle repair, Congenital heart disease, Pulmonary atresia with intact ventricular septum, Left-dominant atrioventricular canal, Left-dominant atrioventriculoseptal defect, Unbalanced atrioventricular canal defect, Unbalanced atrioventriculoseptal defect

## Abstract

**Background:**

Some patients with pulmonary atresia with an intact ventricular septum (PA/IVS) or a left ventricle dominant atrioventricular canal defect (LDAVC) with a hypoplastic right ventricle (RV) and univentricular (1 V) circulation may be candidates for conversion to either a complete biventricular (2 V) repair or a one-and-a-half ventricle repair (1.5 V). We sought to identify pre-operative cardiovascular magnetic resonance (CMR) findings associated with successful conversion from 1V to 1.5V or 2V circulation.

**Methods:**

In this single center retrospective study, subjects with PA/IVS or LDAVC and no conotruncal abnormalities were included if they had a 1 V circulation at the time of CMR followed by a surgical intervention intended to convert them to a 1.5 V or 2 V circulation. Conversion failure was defined as any of the following: (1) oxygen saturation < 90% at the most recent follow-up, (2) conversion back to a 1.5 V or 1 V circulation, or (3) death.

**Results:**

In the PA/IVS cohort (n = 15, median age 1.32 years), 10 patients underwent surgical conversion to a 1.5 V circulation and 5 to a 2 V circulation. In the attempted 1.5 V group, there were 2 failures, and these cases had a lower RV mass (p = 0.04). In the attempted 2 V group, there was 1 failure, and no CMR parameters were significantly different compared to the successes. Among the successful 2 V group patients, the minimum RV end-diastolic volume (EDV) was 27 ml/m^2^. In the LDAVC cohort (n = 15, median age 1.0 years), 1 patient underwent surgical conversion to a 1.5 V circulation and 14 patients to a 2 V circulation. In the attempted 1.5 V group, the 1 conversion was a failure and had an RV EDV of 15 ml/m^2^. In the attempted 2 V group, there were 2 failures, and these cases had a smaller RV:LV stroke volume ratio (p = 0.05) and a lower RV ejection fraction (p = 0.05) compared to the successes. Among the successful 2 V group patients, the minimum RV EDV was 22 ml/m^2^.

**Conclusions:**

We identified multiple CMR parameters associated with successful conversion from 1 V circulation to 1.5 V or 2 V circulation in patients with PA/IVS and LDAVC. This information may improve patient selection for conversion procedures and encourage larger studies to better define the role of CMR.

**Supplementary Information:**

The online version contains supplementary material available at 10.1186/s12968-023-00965-6.

## Background

A range of right ventricular underdevelopment may be seen in congenital heart disease. At one end, patients with severe ventricular hypoplasia and/or valve stenosis require a single ventricle (1 V) palliative approach, culminating in a Fontan circulation. Alternatively, patients less severely affected may be suitable candidates for a biventricular (2 V) circulation or a one-and-a-half ventricular (1.5 V) circulation (i.e., a superior cavopulmonary anastomosis, antegrade flow from the right ventricle (RV) to pulmonary arteries, and a small or no atrial septal defect (ASD)). Deciding which management strategy to pursue has major implications for a patient’s long-term health and can be difficult.

This challenge most commonly arises in patients with one of two anatomic diagnoses: (1) pulmonary atresia with an intact ventricular septum (PA/IVS) and (2) left-dominant atrioventricular canal (LDAVC). Choosing a 1 V palliative approach places them at risk for the long-term complications of a Fontan circulation including ventricular dysfunction, protein losing enteropathy, plastic bronchitis, arrhythmias, thromboembolism, and liver failure [1–5]. Alternatively, aggressive pursuit of a 1.5 V or 2 V circulation risks post-operative morbidity, RV systolic and diastolic dysfunction, valvular dysfunction requiring repeated repairs or replacement, and the consequences of systemic venous hypertension.

In such patients with small right hearts, multiple factors are reviewed to determine whether 1.5 V or 2 V circulation can be supported. These include right heart pressure measurements at catheterization and anatomic measurements by echocardiography such as the tricuspid valve annulus z-score, RV to left ventricle (LV) length ratio, RV/LV area ratio, tricuspid valve function, and ratio of tricuspid and mitral valve area [6–11]. Data from cardiovascular magnetic resonance (CMR) has the potential to impact decision-making as well. CMR is the reference standard for quantification of RV volume and blood flow across vessels and valves; and can identify myocardial fibrosis with the late gadolinium enhancement (LGE) technique. We have previously identified CMR parameters associated with successful 2 V conversion in patients with a small left heart [12]. There are, however, no similar reports of CMR predictors in patients with a small right heart. Thus, the aim of this study was to describe pre-operative CMR findings associated with successful conversion from a 1 V circulation to a 1.5 V circulation or 2 V circulation in patients with a hypoplastic RV.

## Methods

### Subjects

A retrospective database review of all patients who underwent a CMR at Boston Children’s Hospital from January 2005 through December 2020 was conducted. Subjects were included if they had the following: (1) PA/IVS or LDAVC, (2) no conotruncal abnormalities (i.e., tetralogy of Fallot, truncus arteriosus, interrupted aortic arch type B, transposition of the great arteries, double-outlet right ventricle, double-outlet left ventricle, and anatomically corrected malposition of the great arteries), and 3) a 1 V circulation at the time of CMR followed by a surgical or catheter intervention intended to convert them to a 1.5 V or 2 V circulation. LDAVC patients were those with endocardial cushion defects and RV hypoplasia and/or more than 50% of the common atrioventricular valve related to the LV. Patient demographics, clinical history, and CMR data were obtained from the medical record. The Boston Children’s Hospital Institutional Review Board approved this study and a waiver of informed consent.

### Outcomes

All available clinical records were reviewed. The surgical or catheter intervention intended to convert them to a 1.5 V or 2 V circulation was defined as a failure for any one of the following: (1) oxygen saturation < 90% at the time of most recent follow-up, (2) conversion from a 2 V circulation back to a 1.5 V or 1 V circulation, or from a 1.5 V circulation back to a 1 V circulation, or (3) death. Additional outcome measures included RV dysfunction at follow-up defined as a qualitative RV function grade of mild or worse on the most recent echocardiogram, and ASD flow direction on the most recent echocardiogram.

### CMR

CMR was performed on a 1.5 T scanner (Philips Achieva, Philips Medical Systems, Best, the Netherlands). The imaging protocol included (1) steady-state free precession cine imaging in an axial plane covering the thorax and in ventricular long- and short-axis planes; (2) a gadolinium-enhanced magnetic resonance angiogram; (3) phase velocity flow measurements in the ascending aorta, pulmonary valve, atrioventricular valves, and branch pulmonary arteries; and, in some patients, (4) LGE imaging in ventricular long- and short-axis planes 10–15 min after the administration of contrast.

Ventricular volumes and flow measurements were calculated using commercially available software (cvi42, Circle Cardiovascular Imaging Inc., Calgary, Alberta, Canada) as previously described (Additional file 1: Fig. S1 and Additional file 2: Fig. S2) (13). Of note, RV trabeculations were included within the blood pool segmentation. In LDAVC patients, ventricular volumes were measured by extrapolating the plane of the septum to the base of the heart.

### Statistical analysis

Descriptive statistics are shown as median and range. The Mann–Whitney U-test was used to compare parameters between attempted conversion to 2 V versus 1.5 V circulation and patients with a successful versus unsuccessful conversion from a 1 V circulation. A Fisher exact t-test was used to determine the association of RV LGE with failure in the PA/IVS cohort or the association between 2 categorical variables. A p-value of ≤ 0.05 was considered statistically significant. Statistical calculations were performed using SPSS version 23 (IBM Corporation, USA).

## Results

### Pulmonary atresia with an intact ventricular septum cohort

**Fig. 1:**
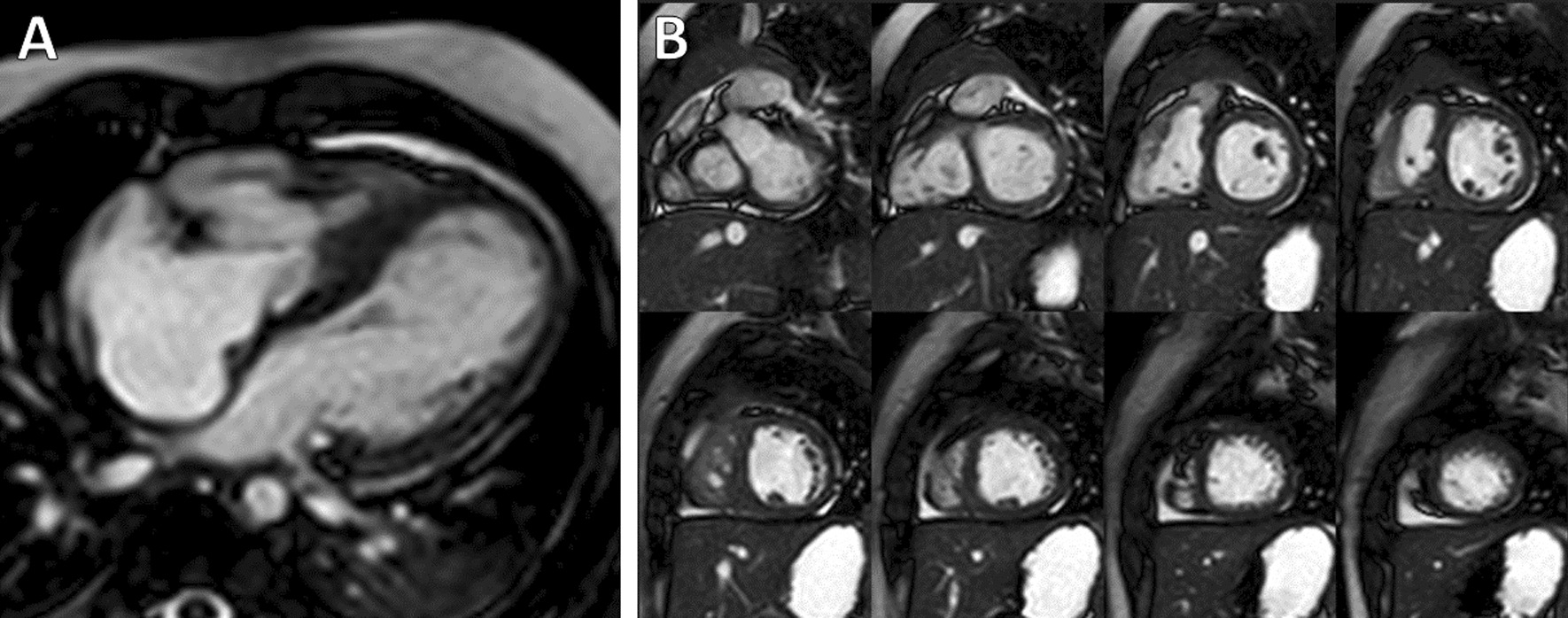
12-month-old male with pulmonary valve atresia and an intact ventricular septum status-post pulmonary valve perforation and dilation and placement of a ductus arteriosus stent at 3 days old. CMR cine images during diastole in 4-chamber **A** and short-axis **B** planes. The patient subsequently underwent a successful biventricular repair surgery

Over the study period, 15 patients with PA/IVS with a 1 V circulation met the inclusion criteria and underwent CMR prior to conversion to a 1.5 V or 2 V circulation (Fig. 1). Their demographic, clinical history, and CMR parameters are summarized in Table 1. Their median age was 1.32 years (0.37–12.63) at CMR and 1.34 years (0.38–13.10) at the conversion procedure. Prior to conversion, 73% of the patients had a dual source of pulmonary flow via modified Blalock-Taussig-Thomas (BTT) shunt, bidirectional Glenn (BDG) shunt, or ductal stent in addition to forward flow across the pulmonary valve; all had a moderate or large ASD with right-to-left flow. Ten patients underwent surgery to convert to a 1.5 V circulation and 5 patients underwent surgery to convert to a 2 V circulation; there were no catheter-based conversion procedures. None of the patients were in both the 1.5 V and the 2 V conversion groups. The only CMR parameter that was associated with attempted conversion to a 2 V rather than 1.5 V circulation was a larger RV end-diastolic volume (EDV) (p = 0.03).

**Table 1:** Demographic and CMR findings of PA/IVS and LDAVC cohorts

	PA/IVS (n = 15)			LDAVC (n = 15)		
	All	1.5 V	2 V	All	1.5 V	2 V
N	15	10	5	15	1	14
Age at CMR (yrs)	1.32 (0.37–12.63)	3.27 (0.37–12.63)	1.07 (0.47–1.32)	1.04 (0.20–5.20)	0.40	1.13 (0.20–5.20)
Age at conversion surgery (yrs)	1.34 (0.38–13.10)	3.84 (0.38–13.10)	1.10 (0.48–1.43)	1.24 (0.22–5.20)	0.46	1.30 (0.22–5.20)
Female (n)	5	3	2	11	0	11
Genetic syndrome (n)	1	1	0	15	1	14
Body surface area (m^2^)	0.50 (0.30–1.35)	0.55 (0.30–1.35)	0.46 (0.33–0.50)	0.39 (0.25–0.68)	0.31	0.40 (0.25–0.68)
Circulation at CMR (n)						
Modified BTT/central shunt	3	1	2	3	1	2
Pulmonary artery band	0	0	0	8	0	8
Bidirectional Glenn	6	6	0	2	0	2
Ductal stent	5	2	3	0	0	0
Fontan	1	1	0	0	0	0
Dual source of pulmonary blood flow (n)	11	6	5	2	0	2
CMR parameters						
Heart rate (bpm)	106 (76–135)	104 (76–126)	125 (101–135)	110 (71–130)	130	106 (71–125)
RV EDV (ml/m^2^)	34 (13–55)	24 (13–40)	46 (27–55)	41 (15–102)	15	43 (22–102)
RV ESV (ml/m^2^)	13 (6–27)	13 (6–20)	13 (10–27)	14 (7–64)	7	15 (8–64)
RV stroke volume (ml/m^2^)	19 (2–33)	6 (2–31)	30 (14–33)	27 (8–55)	8	28 (13–55)
RV ejection fraction (%)	54 (10–78)	53 (10–78)	54 (43–74)	61(37–75)	56	62 (37–75)
RV mass (g/m^2^)	10 (5–21)	7 (5–15)	12 (5–21)	15 (5–33)	5	16 (7–33)
LV EDV (ml/m^2^)	85 (51–166)	85 (59–127)	75 (51–166)	97 (55–250)	95	98 (55–250)
LV ESV (ml/m^2^)	36 (16–88)	36 (21–61)	28 (16–88)	36 (23–123)	42	35 (23–123)
LV stroke volume (ml/m^2^)	49 (27–78)	50 (36–70)	48 (27–78)	60 (29–128)	52	62 (29–128)
LV ejection fraction (%)	60 (44–73)	59 (44–71)	60 (47–73)	60 (51–70)	55	61 (51–70)
RV:LV stroke volume ratio	0.41 (0.04–0.70)	0.17 (0.04–0.54)	0.51 (0.26–0.70)	0.45 (0.15–0.81)	0.15	0.50 (0.21–0.81)
TV:MV inflow ratio	0.18 (0.004–0.51)	0.18 (0.004–0.27)	0.42 (0.12–0.51)			
TV anteroposterior diameter (cm)	1.35 (0.44–1.70)	0.98 (0.44–1.70)	1.40 (1.18–1.62)			
TV lateral diameter (cm)	1.23 (0.47–1.80)	1.10 (0.47–1.80)	1.60 (1.35–1.61)			
RV LGE present / LGE performed (n)	4/11	2/7	2/4	0/10	0/1	0/9

Values are median (range) unless specified as counts (n). *BTT* Blalock-Taussig-Thomas, *CMR* cardiac magnetic resonance, *EDV* end-diastolic volume, *ESV* end-systolic volume, *LDAVC* left-dominant atrioventricular canal, *LGE* late gadolinium enhancement, *LV* left ventricle, *MV* mitral valve, *PA/IVS* pulmonary atresia with an intact ventricular septum, *RV* right ventricle, *TV* tricuspid valve

In the attempted 1.5 V group (n = 10), over a median post-surgical follow-up time of 40 months (18–75), there were 2 failures (Table 2). One patient failed because of an oxygen saturation < 90%. The other patient underwent a Fontan procedure 2 years after their 1.5 V conversion surgery because of significant hypoxia. The only pre-operative CMR parameter associated with a successful 1.5 V conversion was a larger RV mass (p = 0.04) (Table 2). Among the successful 1.5 V group (n = 8), the minimum RV EDV was 15 ml/m^2^. Over a median post-surgical follow-up time of 40 (18–75) months, 4 of the 8 patients underwent a subsequent surgery (branch pulmonary artery plasty (n = 1); pulmonary valve replacement, tricuspid valve plasty, and division of RV muscle bundles (n = 1); pulmonary valve replacement, tricuspid valve plasty, and creation of small atrial septal fenestration (n = 1); and tricuspid valve plasty and right atrial reduction (n = 1)), but all maintained a successful 1.5 V circulation. Also, 2 of the 8 patients underwent catheter interventions (stenting of atrial septum (n = 1) and pulmonary valve balloon dilatation followed 6 months later by transcatheter pulmonary valve replacement (n = 1)).

**Table 2:** Outcomes of PA/IVS and LDAVC cohorts

	PA/IVS						LDAVC			
	1.5 V (n = 10)			2 V (n = 5)			1.5 V (n = 1)	2 V (n = 14)		
	Success	Failure	p-value	Success	Failure	p-value	Failure	Success	Failure	p-value
N	8	2		4	1		1	12	2	
CMR parameters										
RV EDV (ml/m^2^)	24 (15–40)	23 (13–34)	0.29	43 (27–55)	48	0.76	15	48 (22–102)	28 (22–35)	0.13
RV ESV (ml/m^2^)	13 (6–20)	11 (6–15)	0.36	13 (10–25)	27	0.92	7	16 (8–64)	12 (8–16)	0.17
RV stroke volume (ml/m^2^)	11 (2–31)	13 (6–19)	0.38	30 (14–33)	20	0.24	8	30 (13–55)	17 (14–19)	0.07
RV ejection fraction (%)	41 (10–78)	53 (49–57)	0.39	63 (52–74)	43	0.08	56	62 (55–66)	60 (55–66)	0.05
RV mass (g/m^2^)	13 (9–21)	7 (6–9)	0.04	6 (5–14)	15	0.14	5	17 (7–33)	10 (8–12)	0.07
LV EDV (ml/m^2^)	84 (59–127)	111 (108–15)	0.11	67 (51–104)	166	0.14	94	98 (55–251)	96 (77–115)	0.37
LV ESV (ml/m^2^)	35 (21–61)	52 (45–60)	0.11	26 (16–41)	88	0.14	42	35 (23–123)	36 (25–48)	0.36
LV stroke volume (ml/m^2^)	49 (36–66)	59 (47–70)	0.34	45 (27–62)	78	0.92	52	62 (29–128)	60 (52–67)	0.42
LV ejection fraction (%)	59 (52–71)	53 (44–61)	0.34	62 (53–73)	47	0.92	55	61 (51–70)	63 (59–68)	0.16
RV:LV stroke volume ratio	0.36 (0.08–0.55)	0.25 (0.09–0.41)	0.29	0.60 (0.47–0.70)	0.26	0.14	0.16	0.58 (0.21–0.82)	0.28 (0.27–0.28)	0.05
TV inflow (L/min/m^2^)	0.40 (0.02–0.99)	0.54 (0.01–1.06)	0.39	1.64 (0.72–1.91)	1.03	0.31				
MV inflow (L/min/m^2^)	2.00 (1.66–4.15)	2.98 (2.13–3.83)	0.37	4.18 (2.97–5.28)	6.26	0.15				
TV:MV inflow ratio	0.18 (0.01–0.23)	0.14 (0.005–0.28)	0.35	0.44 (0.14–0.51)	0.12	0.14				
TV AP diameter (cm)	1.10 (0.44–1.70)	0.98	0.39	1.45 (1.18–1.62)	1.40	0.40				
TV lateral diameter (cm)	1.15 (0.47–1.80)	0.80	0.38							
TR ≥ mild (n)	4	1	1.0	2	1	1.0				
RV LGE present / LGE performed (n)	1/5	1/2	0.52	1/3	1/1	1.0	0	0/9	0/1	
Follow-up (months)	40 (18–75)	35 (26–44)		57 (1.4–95)	71		76	45 (0.5–116)	1.6 (0.5–2.6)	
Subsequent surgery (n)	4	1		1	0		0	2	0	
Subsequent catheterization intervention (n)	2	1		0	1		1	3	0	
Death (%)	0	0		0	0		0	0	0	
Symptomatic (%)	2	1		3	0		0	2	0	
Saturation at last follow-up (%)	98 (96–100)	92 (89–95)		100 (95–100)	88		88	98 (92–100)	88 (88–89)	
RV dysfunction (n)	2	1		2	0		0	0	0	
ASD flow										
None (n)	5	1		3	0		0	4	0	
Left-to-right (n)	1	0		0	0		0	4	0	
Bidirectional (n)	1	0		0	0		0	1	2	
Right-to-left (n)	1	1		1	1		1	2	0	

Values are median (range) unless specified as counts (n). *CMR* cardiac magnetic resonance, *EDV* end-diastolic volume, *ESV* end-systolic volume, *LDAVC* left-dominant atrioventricular canal, *LGE LGE* late gadolinium enhancement, *LV* left ventricle, *MV* mitral valve, *PA/IVS* pulmonary atresia with an intact ventricular septum, *RV* right ventricle, *TR* tricuspid regurgitation, *TV* tricuspid valve

In the attempted 2 V group (n = 5), over a median post-surgical follow-up time of 60 months (1.4–95), there was 1 failure (Table 2). This patient underwent a BDG for progressive cyanosis and exercise intolerance 6 years after his 2 V conversion. Among the successful 2 V group (n = 4), the minimum RV EDV was 27 ml/m^2^. Over a median post-surgical follow-up time of 57 (1.4–95) months, 1 of the 4 patients underwent a subsequent surgical intervention (surgical pulmonary valve replacement and tricuspid valve plasty).

### Left-dominant atrioventricular canal defect cohort

**Fig. 2:**
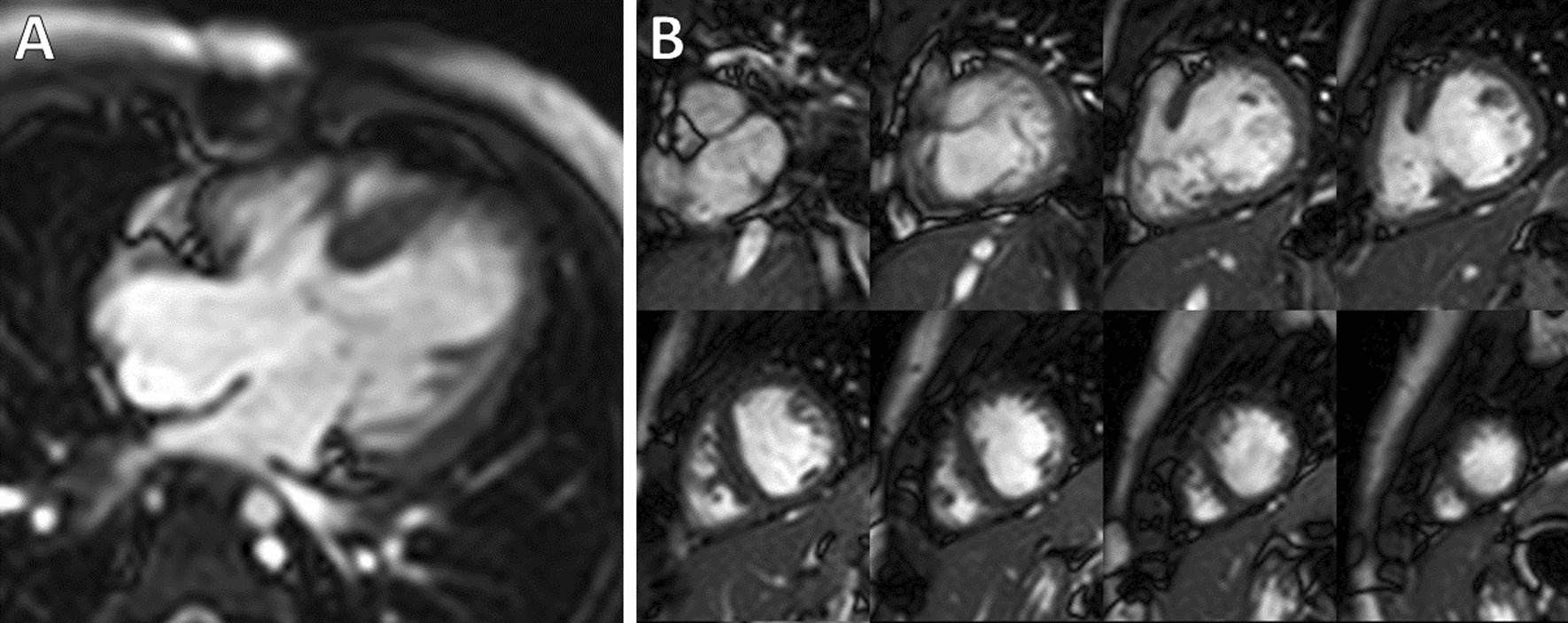
5-year-old female with trisomy 21 and a left-dominant complete atrioventricular canal defect status-post pulmonary artery band placement at age 2 months and status-post a bidirectional Glenn shunt and over-sewing of the main pulmonary artery at age 11 months. CMR cine images during diastole in 4-chamber **A** and short-axis **B** planes. The patient subsequently underwent a successful biventricular repair surgery

Over the study period, 15 patients with a LDAVC and a 1 V circulation met the inclusion criteria and underwent CMR prior to conversion to a 1.5 V or 2 V circulation (Fig. 2). Their demographic, clinical history, and CMR parameters are summarized in Table 1. Of note, all of the patients in this cohort had trisomy 21. Their median age was 1.04 year (0.20–5.20) at CMR and 1.24 years (0.22–5.20) at the conversion procedure. Prior to conversion, only 13% of the patients had a dual source of pulmonary flow via modified BTT shunt or BDG in addition to forward flow across the pulmonary valve; all had a moderate or large ASD. One patient underwent surgery to a convert to a 1.5 V circulation and the other 14 patients underwent surgery to convert to a 2 V circulation. There were no catheter-based conversion procedures. The small number of conversions to a 1.5 V circulation precluded a meaningful comparison between the attempted conversion to a 1.5 V versus 2 V circulation groups.

The 1 patient in the attempted 1.5 V conversion group underwent a BDG, pulmonary artery plasty, right ventricular outflow tract patch, and ASD closure with a fenestrated patch. Over a post-surgical follow-up time of 76 months, his only intervention was a left pulmonary artery stent placement during a catheterization 17 months after the conversion surgery (Table 2). His most recent oxygen saturation was 88% meeting criteria for failure. Of note, his RV EDV was 15 ml/m^2^, the smallest among the entire LDAVC cohort.

In the attempted 2 V surgical conversion group (n = 14), over a median post-surgical follow-up time of 39 months (0.5–116), there were 2 failures, both for oxygen saturation < 90% (specifically, 88% and 89%) (Table 2). Neither had a subsequent surgery or catheterization intervention. A successful versus failed 2 V conversion was associated with a higher pre-operative CMR RV ejection fraction (p = 0.05) and a larger RV:LV stroke volume ratio (p = 0.05). There was also a trend toward a higher RV mass (p = 0.07) and higher RV stroke volume (p = 0.07) in the successful group. Among the successful 2 V group (n = 12), the minimum RV EDV was 22 ml/m^2^. Over a median post-surgical follow-up time of 45 months (0.5–116), 2 of the 12 successful 2 V patients underwent subsequent surgery. One patient underwent pacemaker placement 9 days after 2 V conversion surgery. The second patient underwent (1) ASD closure for patch dehiscence and cyanosis 7 days after surgery, (2) tricuspid valve repair for insufficiency 18 months after conversion surgery, and 3) tricuspid valve replacement with a Melody valve for stenosis, ASD creation, and RV muscle bundle resection 19 months after conversion surgery. Another 3 of the 12 patients underwent catheter interventions following conversion surgery (ASD device closure (n = 1), balloon dilatation of a pulmonary vein (n = 1), and balloon dilatation of the pulmonary valve (n = 1)).

## Discussion

This study is the first to report CMR descriptive data and findings associated with successful conversion from 1 V circulation to 1.5 V or 2 V circulation in patients with right heart hypoplasia. In patients with PA/IVS, a greater RV mass was associated with successful conversion to a 1.5 V circulation and none of the CMR parameters were significantly associated with successful conversion to a 2 V circulation. The presence of RV LGE was not associated with successful conversion. A successful 2 V conversion was seen with an RV EDV as low as 27 ml/m^2^. In patients with a LDAVC, a larger RV:LV stroke volume ratio and a higher pre-operative CMR RV ejection fraction were associated with successful conversion to 2 V circulation, and a success was seen with an RV EDV as low as 22 ml/m^2^. These results may aid decision-making and serve as a stimulus for further study.

Patients with significant right heart hypoplasia often pose a management dilemma. A 1 V palliative approach incurs the morbidity and mortality risks of a Fontan circulation, and these risks tend to increase as patients age. Conversion to a 1.5 V or 2 V circulation may reduce these risks, particularly those that are related to elevated systemic venous pressure. Moreover, having a ventricle propel all or part of the systemic venous return to the pulmonary circulation may improve cardiovascular performance [14]. However, overly ambitious attempts to achieve a 1.5 V or 2 V circulation may lead to systemic venous hypertension from tricuspid stenosis or elevated RV filling pressure as seen in some of our study patients and thereby thwart one of the main objectives of the conversion procedure.

Management decisions in patients with right heart hypoplasia are often made by integrating multiple patient factors with a strong reliance on data from echocardiography and cardiac catheterization. For example, relevant echocardiographic measurements in unbalanced atrioventricular canals include the atrioventricular valve index [15], RV/LV inflow angle [16], and right and left ventricular size and function [9]. In PA/IVS, tricuspid valve annular size, tricuspid valve function, and RV size and systolic function are important. Nevertheless, room remains for improvement in the decision-making algorithms and perhaps for the identification of more patients who would benefit from a 1.5 V or 2 V circulation approach. The use of CMR parameters has the potential to contribute to this effort as it is the reference standard for measuring RV volume and systolic function, blood flow across vessels and valves, and myocardial fibrosis that may contribute to ventricular diastolic dysfunction.

Despite its small sample size, our study was able to identify CMR parameters that were associated with a successful conversion from 1 V circulation to 1.5 V or 2 V circulation. In patients with a LDAVC, a larger RV:LV stroke volume ratio and a higher pre-operative CMR RV ejection fraction were linked with successful conversion to 2 V repair. Both of these findings make physiological sense as the RV is required to handle a greater volume of blood with the conversion procedure. Based on this data, we would strongly consider a 2 V conversion if the RV:LV stroke volume ratio is ≥ 0.4 and the RV EF is ≥ 55%. In patients with PA/IVS, a greater RV mass was associated with successful conversion to a 1.5 V circulation. Greater mass is likely advantageous because it allows the RV to maintain a higher mass:volume ratio in the setting of a volume increase. An insufficient mass to volume ratio leads to increased wall stress and afterload which may predispose the ventricle to systolic dysfunction. Our data suggest that patients with a RV mass less than approximately 10 g/m^2^ should not be considered for a 1.5 V or 2 V conversion. Moreover, in both the PA/IVS and LDAVC groups, some patients with moderately hypoplastic RVs were still able to achieve a successful 2 V conversion. This suggests that their RVs are relatively compliant and can adapt to an increase in inflow without a dramatic rise in filling pressure. Thus, an RV EDV as low as approximately 30 ml/m^2^ should not preclude an attempt at a 2 V conversion if other factors are favorable. Lastly, there were 4 patients with PA/IVS and an ejection fraction < 45% that were able to achieve a successful 1.5 V conversion. The impact of conversion on RV ejection fraction and the benefits of a 1.5 V circulation versus a Fontan circulation require further study.

## Limitations

Our study has several important limitations. It had a relatively small sample size as PA/IVS and LDAVC are rare conditions and only a subset of patients with these diagnoses underwent a 1.5V or 2V conversion procedure. Moreover, our study design excluded patients who had a CMR study but did not undergo a conversion procedure, perhaps based on the CMR data itself. Both of these factors could have potentially decreased the power of the study to identify CMR parameters associated with a successful conversion. Moreover, all patients underwent their CMR studies and conversion procedures at a single institution thereby limiting the study’s generalizability. Lastly, this study did not have long-term follow-up on patients because many of the conversion surgery procedures were relatively recent. It is possible that over time additional patients may meet the criteria for failure.

## Conclusion

This study identified multiple CMR parameters associated with successful conversion from 1 V circulation to 1.5 V or 2 V circulation in patients with PA/IVS and LDAVC. Moreover, it showed that successful 2 V conversions could be achieved in PA/IVS patients with an RV EDV as low as 27 ml/m^2^ and in LDAVC patients with an RV EDV as low as 22 ml/m^2^. More studies that incorporate additional centers and subjects and with longer follow-up are needed to improve patient selection for conversion procedures and better define the role of CMR.

### Supplementary Information

**Additional file 1: Figure S1.** 12-month-old male with pulmonary valve atresia and an intact ventricular septum status-post pulmonary valve perforation and dilation and placement of a ductus arteriosus stent at 3 days old. CMR cine images at end-diastole in the ventricular short-axis plane illustrating ventricular contours.

**Additional file 2: Figure S2.** 5-year-old female with trisomy 21 and a left-dominant complete atrioventricular canal defect status-post pulmonary artery band placement at age 2 months and status-post a bidirectional Glenn shunt and over-sewing of the main pulmonary artery at age 11 months. CMR cine images at end-diastole in the ventricular short-axis plane illustrating ventricular contours.

## Data Availability

The datasets used and/or analyzed during the current study are available from the corresponding author on reasonable request.

## Abbreviations

1 V: Univentricular
1.5 V: One-and-a-half ventricular
2 V: Biventricular
ASD: Atrial septal defect
BDG: Bidirectional Glenn
BTT: Blalock-Taussig-Thomas
CMR: Cardiovascular magnetic resonance
EDV: End-diastolic volume
LGE: Late gadolinium enhancement
LDAVC: Left-dominant atrioventricular canal
LV: Left ventricle
PA/IVS: Pulmonary atresia with an intact ventricular septum
RV: Right ventricle
