# Supplementary figures and images for: Cardiac magnetic resonance parameters associated with successful conversion from a single ventricular to a one-and-a-half or biventricular circulation in patients with a hypoplastic right ventricle
Source: J Cardiovasc Magn Reson. 2023 Sep 28;25:51. doi: 10.1186/s12968-023-00965-6 (PMC10537142; doi:10.1186/s12968-023-00965-6)

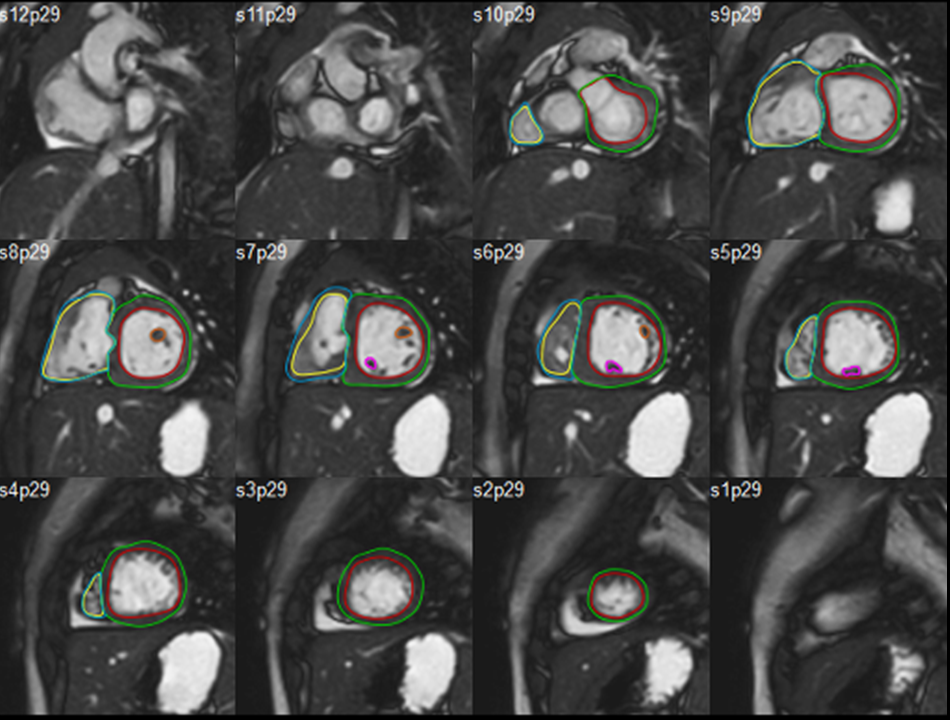

Supplement: Supplementary file 1 — Additional file 1: Figure S1. 12-month-old male with pulmonary valve atresia and an intact ventricular septum status-post pulmonary valve perforation and dilation and placement of a ductus arteriosus stent at 3 days old. CMR cine images at end-diastole in the ventricular short-axis plane illustrating ventricular contours. [file 12968_2023_965_MOESM1_ESM.png]

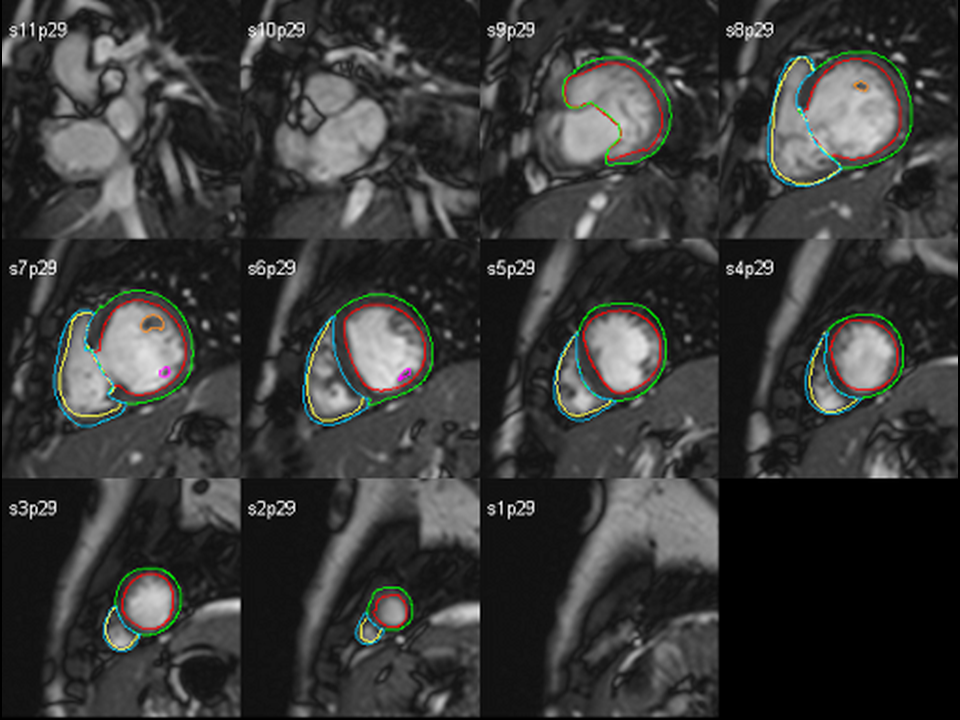

Supplement: Supplementary file 2 — Additional file 2: Figure S2. 5-year-old female with trisomy 21 and a left-dominant complete atrioventricular canal defect status-post pulmonary artery band placement at age 2 months and status-post a bidirectional Glenn shunt and over-sewing of the main pulmonary artery at age 11 months. CMR cine images at end-diastole in the ventricular short-axis plane illustrating ventricular contours. [file 12968_2023_965_MOESM2_ESM.png]
